# 2000 Years of Pandemics: Past, Present, and Future

**DOI:** 10.3201/eid3102.240798

**Published:** 2025-02

**Authors:** Nkuchia M. M’ikanatha, Keith Hamilton

**Affiliations:** Pennsylvania Department of Health, Harrisburg, Pennsylvania, USA (N.M. M’ikanatha); University of Pennsylvania, Philadelphia, Pennsylvania, USA (K. Hamilton)

**Keywords:** outbreaks, communicable diseases, epidemiology, microbiology, humans, pandemics, public health surveillance, COVID-19, respiratory infections, severe acute respiratory syndrome coronavirus 2, SARS-CoV-2, SARS, coronavirus disease, coronavirus, HIV/AIDS and other retroviruses, bacteria, influenza, viruses, zoonoses, book review

In *2000 Years of Pandemics: Past, Present, and Future*, the authors expertly describe the microbiologic origins and dire consequences of contagions when they spread through global populations (Figure). The authors bring diverse experiences to their work. Dr. Ferreira, a physician-scientist in France, is engaged in responses to emerging pathogens, whereas Drs. Doursout and Balingit are both medical educators in the United States. They argue that pandemics are not random events but are the direct result of imbalances created by humanity’s interactions with the environment. As mobility has evolved from the Silk Road to air travel, dangerous microbes have found new, increasingly efficient ways to spread globally. The book includes a timely discussion of how climate change might exacerbate pandemic emergence.

**Figure:**
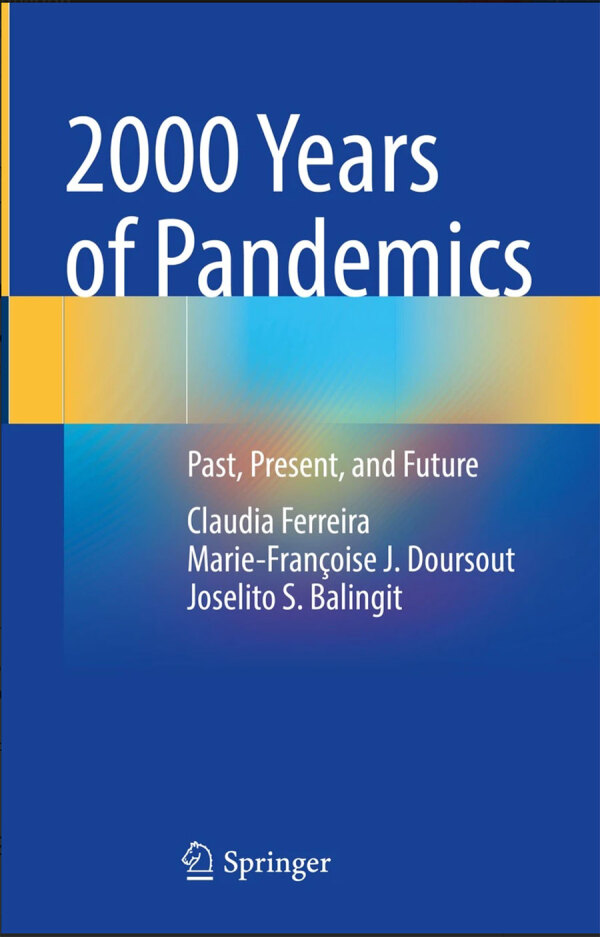
2000 Years of Pandemics: Past, Present, and Future

The authors catalog pandemics starting in 165 CE, thoroughly describing most likely etiologies, effects on contemporary societies, and human responses. The accounts provide vivid and engaging descriptions of what it must have been like to go through those times. The book suggests parallels among pandemics over millennia. Even the vignettes in the pre–germ theory era offer valuable takeaways. During the bubonic plague in Italy, Venetians implemented quarantine and isolation measures, which lowered incidence rates in the city. Those practices remain crucial in infection prevention and control today. Plague doctors donned one of the first and most elaborate forms of personal protective equipment, complete with masks with glass red eyes and birdlike beaks packed with aromatic herbs and spices thought to filter and purify the air they inhaled. Their bodies were almost completely sealed off from environmental contact. The eerie ensemble resembles modern air-purifying respirators and layered protective gear worn by healthcare workers treating Ebola patients. The authors draw parallels between the stigmatization of the homosexual community during the HIV/AIDS pandemic and the misdirected xenophobia and discrimination witnessed in the past. The book also illuminates the horrific history of using disease as a weapon, referencing the gruesome tactic of the Mongol army hurling plague victims’ remains into Caffa, Italy, and the intentional spread of smallpox among Native Americans.

Consistency and organization are the main shortcomings of the book. Some sections read like a compelling history text and others a medical textbook with extensive treatment tables listing antibiotic choice, dose, and duration, specific guidelines for vaccination, and detailed options for diagnostic tests. This level of detail is inconsistent and unnecessary, leading to outdated treatment tables. The authors try to cover too many issues, thus, providing superficial reviews of some topics. In our view, a clearly defined philosophy and a consistent analytical framework would have enhanced this work. Such a framework would have resulted in deeper explorations of each pandemic, drawing out valuable insights from societal responses and their implications for future pandemics.

The book is engaging and instructive offering insights for a wide range of audiences, including biomedical trainees and practitioners and policymakers actively involved in pandemic responses. Published in 2023, this book uniquely connects the responses to and effects of pandemics, drawing parallels and extracting valuable lessons. It provides crucial observations into how pandemics have shaped surveillance, preparedness, and treatment of infectious diseases, and how they will continue to influence human history.

